# Discontinuous Dissolution Reaction in a Fe-13.5 at. % Zn Alloy

**DOI:** 10.3390/ma14081939

**Published:** 2021-04-13

**Authors:** Paweł Zięba, Mateusz Chronowski, Jarosław Opara, Olga A. Kogtenkova, Boris B. Straumal

**Affiliations:** 1 Institute of Metallurgy and Materials Science, Polish Academy of Sciences, 30-059 Cracow, Poland; m.chronowski@imim.pl; 2 Łukasiewicz Research Network–Institute for Ferrous Metallurgy, 44-100 Gliwice, Poland; jaroslaw.opara@imz.pl; 3 Institute of Solid State Physics, Russian Academy of Sciences, 142432 Chernogolovka, Russia; koololga@issp.ac.ru (O.A.K.), straumal@mf.mpg.de (B.B.S.)

**Keywords:** discontinuous dissolution, diffusion at moving boundaries, solute concentration profiles, TEM/EDX microanalysis, Fe-Zn system

## Abstract

The dissolution process of a lamellar structure with α and Γ phases formed during a discontinuous precipitation reaction is investigated here with a Fe-13.5 at. % Zn alloy by means of optical microscopy and scanning and transmission electron microscopy. The α phase is a solute-depleted solid solution and the Γ phase is the intermetallic compound Fe_3_Zn_10_. The examination reveals that the dissolution occurs in a discontinuous mode by a receding of the former reaction front of the discontinuous precipitation towards the position of the original grain boundary. A new solid solution in the post-dissolution area is especially inhomogeneous and reflects the former locations of the Γ lamellae (“ghost images”) and the receding reaction front (“ghost lines”). A simulation procedure is applied to determine the Zn concentration profiles left in the post-dissolution region. Their shapes are mostly affected by the Zn content at the positions where the Γ lamellae have just been dissolved, which was also confirmed by the quantitative microchemical analysis.

## 1. Introduction

Fe-Zn systems represent a very well described example [1,2,3,4] in which the decomposition of a α Fe-rich supersaturated solid solution occurs via discontinuous precipitation (DP). Moreover, subsequent annealing above a certain temperature results in a reverse process, discontinuous dissolution (DD), which is characterized by a backward migration of the DP front (i.e., towards the original position of the grain boundary) with a simultaneous formation of an inhomogeneous α~ solid solution [3,5]. 

The DD reaction was investigated by Nakkalil and Gupta [5] in a Fe-17 at. % Zn alloy. The process started and occurred at the primary grain boundaries, impingements of the colonies and at the primary cells with the contact with untransformed regions. The kinetics of the process were analysed using a Petermann-Hornbogen equation [6] with the conclusion that the dissolution of the cells occurs via the diffusion of Zn along the receding reaction front (RF). Similar conclusions were taken by Chuang et al. [3] in a Fe-13 at. % Zn alloy. Additionally, they documented the so-called go- and -stop motion of the receding colonies of discontinuous precipitates using scanning electron microscopy. 

The recent studies [7] have shown the potential of analytical electron microscopy for the determination of solute concentration profiles left behind a moving RF of a discontinuous precipitate in a Fe-13.5 at. % Zn alloy. These profiles are converted into the grain boundary diffusivity values, indicating that the rate controlling factor for the DP reaction in this alloy is diffusion at the moving RF.

The present study is undertaken with two objectives in mind. The first one is to describe the occurrence of discontinuous dissolution in the Fe-13.5 at. % Zn alloy in terms of microstructural changes. Especially, reasonable explanation of the go- and -stop movement is still needed. One should note that the Fe-Zn system shows the DP and DD reactions in a relative range of temperatures and compositions. It also attracts attention due to the well-known process of steel zincification. The second objective arises from the fact that by possessing data for the DP process (see [7]) we can make the next step in the description of discontinuous phase transformations in the Fe-13.5 at. % Zn alloy and predict the changes in chemical composition accompanying the discontinuous dissolution and compare them with the experiment. This in turn should help us in better understanding the rate of diffusion processes occurring at the moving high-angle boundaries in the case where the resulting solid solution is especially inhomogeneous. 

## 2. Materials and Methods

A Fe-13.5at. % Zn alloy was prepared using iron (99.8% purity) and zinc (99.99% purity) in an autoclave under an argon atmosphere of 1.5 GPa. Rods (10 mm × 9 mm in diameter) were subjected to thermal cycling at 723 K for 40 h followed by 5 h at 1073 K in quartz capsules under a vacuum of 10 Pa in order to reduce the grain size down to 50–250 μm. The rods were cut into slices of 1.5 mm and aged in an evacuated silica capsule at 723 K followed by 925 K to develop the discontinuous dissolution. 

Samples for the metallographic observation using light microscopy (LM) and scanning electron microscopy (SEM) were prepared by grinding and then polishing with 0.05 μm Al_2_O_3_. In order to observe the structure after the DP and DD reactions, the samples were etched using a solution of 10% HNO_3_ in methanol for 10 to 15 s.

Further microstructure observations were carried out via scanning electron microscopy (Philips Xl30, Philips Electron Optics B.V., Eindhoven, The Netherlands and FEI E-SEM XL30, FEI, Hillsboro, OR, USA) using the same etched samples for the LM examination. The most demanding research was performed using transmission electron microscopy (Philips CM 20 Twin and Tecnai G2 FEG Super-Twin, FEI, Hillsboro, OR, USA), where the Tecnai instrument was equipped with an EDAX Phoenix energy-dispersive X-ray spectrometer (EDX) (FEI, Hillsboro, OR, USA), enabling high spatial resolution chemical analysis of the solute content in the DD products. 

The foils for the transmission electron microscope (TEM) studies were prepared from slices mechanically thinned to 0.25 mm from which discs that were 3 mm in diameter were cut using spark erosion. The final operation was dimpling and ion beam thinning using a GATAN Duomill instrument (GATAN, Pleasanton, CA, USA). Some thin foils were also prepared from the carefully selected regions close to the RF of discontinuous dissolution using a Quanta 3D focused ion beam (FIB).

## 3. Results and Discussion

**Figure 1:**
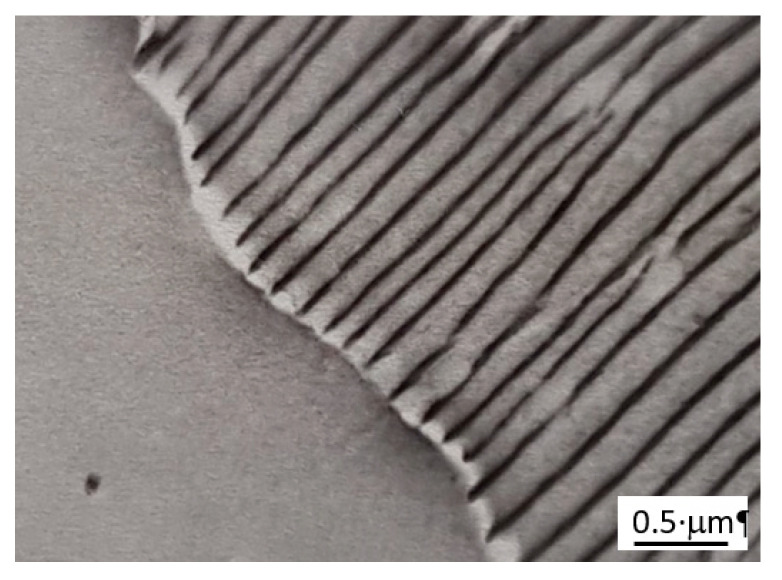
SEM micrograph showing a lamellar microstructure colony of α and Γ phases in Fe-13.5 at. % Zn alloy aged at 723 K for 5 h.

Figure 1 presents a SEM micrograph image of the Fe-13.5 at. % Al alloy aged at 723 K for 5 h. It is visible that the process has already attained a steady state period of growth which is manifested by a co-operative movement of nearly parallel and alternating lamellae which were identified previously [7] as the α (bcc Fe-based solid solution) and Γ (Fe_3_Zn_10_) phases according to Fe-Zn equilibrium phase diagram [8].

**Figure 2:**
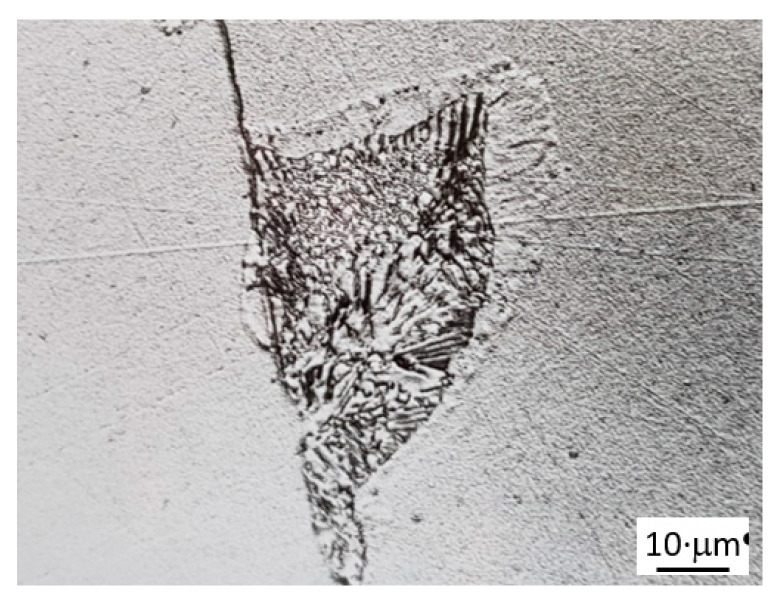
Light microscopy (LM) micrograph showing dissolution at the reaction front of the primary single colony. Fe-13.5 at. % Zn alloy aged at 723 K for 2.5 h to obtain discontinuous precipitation (DP) and annealed at 925 K for 10 min.

A subsequent annealing of such microstructure at 925 K leads to the DD of the lamellar structure. Figure 2 shows a single colony of discontinuous precipitates after receding from its original position by the distance of approximately 10 μm in the central part.

**Figure 3:**
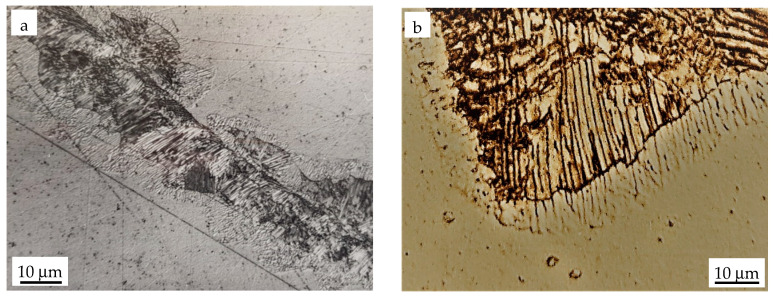
LM micrographs of “ghost images” after dissolution of the discontinuous precipitates in Fe-13.5 at. % Zn alloy aged at 723 K for 2.5 h to obtain DP and annealed at 925 K for 10 min. (**a**)overall view, (**b**) receding reaction font at higher magnification.

Figure 3a shows the DD process in a more advanced stage over a relatively large area of discontinuous precipitates. After such time of annealing, the first symptoms of DD are also visible at the prior grain boundary. The contrast visible in the dissolved area (Figure 2 and Figure 3a) shows good evidence that the solid solution formed due to this process is especially inhomogeneous.

This is clearly shown in Figure 3b, which depicts a light microscopy micrograph obtained by applying a special contrast for better revealing the peculiar lines in the dissolved area, reflecting positions where the Fe_3_Zn_10_ lamellae previously existed. These images are referred to as “ghost images”. It should be emphasized that if time of sample etching is too short or the etchant is not aggressive enough, then the dissolved areas appear in the form of white and uniform slabs or islands, which brings misleading information that the newly formed solid solution is rather homogeneous. Such an image was attributed by Nakkalil and Gupta [5] for a longer time of dissolution after which the composition in the dissolved area became homogeneous; however, in light of the present study, this is not true as “ghost images” are always present. They could be removed by volume diffusion but this process would take rather days than few minutes. Contrary to Sulonen [9], who claimed that only complete dissolution results in a ghosted structure, we observed it at each stage of dissolution.

**Figure 4:**
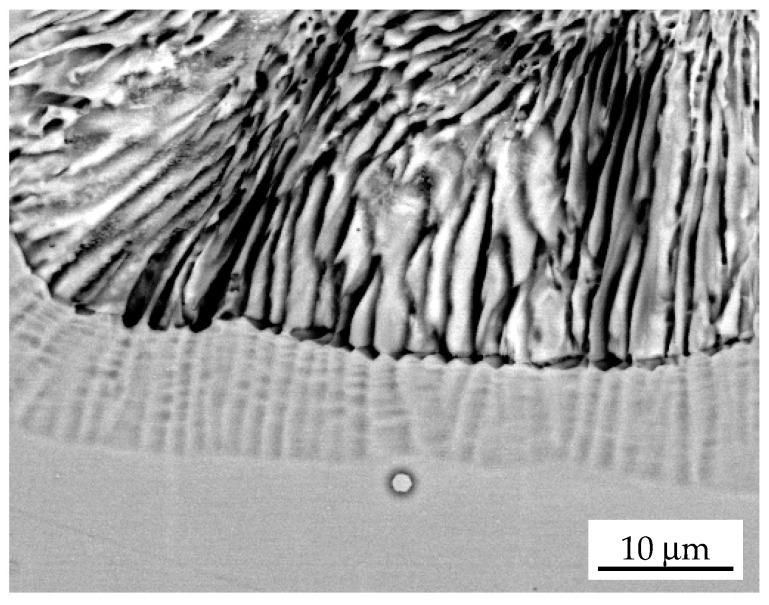
SEM micrograph showing dissolution of the discontinuous precipitates in Fe-13.5 at. % Zn alloy aged at 723 K for 2.5 h to obtain DP and annealed at 925 K for 2 min.

The “ghost images” are better visible using scanning electron microscopy (Figure 4). Not only are the previous locations the Γ phase lamellae present, but also the successive positions of the receding RF of discontinuous dissolution, which forms the characteristic “dotted” lines that parallel to each other. This also means that the backward movement of the RF is not continuous and occurs in the so-called go- and -stop fashion. This is exactly the same mechanism which has recently been reported for a DP reaction in an AlZn alloy [10], but it occurs in a reverse direction. The Zn atoms leave the tip of the Γ phase lamella by diffusion along the RF towards the centre of the α lamella; however, not all the atoms are able to diffuse up to a distance of λ_α_/2 during the displacement of the RF by its own width at the certain velocity value. The highest Zn content reflects the former position of dissolved Γ lamella. After some time, the RF is “clogged” with excessive Zn atoms which causes the movement to halt. This stage is visible in the form of the “ghosted” lines parallel to the RF. The stop period enables all the Zn atoms to enter the new α solid solution. The RF is relaxed, and the receding process then starts again. 

**Figure 5:**
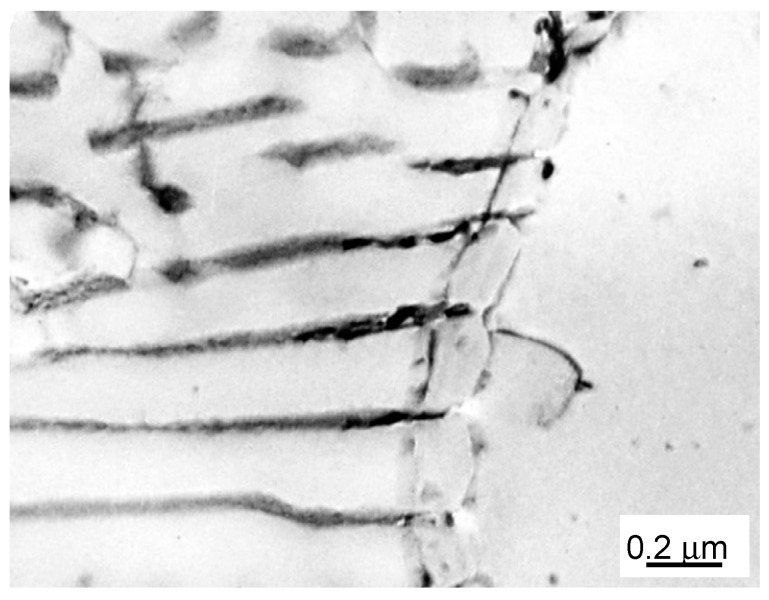
TEM micrograph showing dissolution of the discontinuous precipitates in Fe-13.5 at. % Zn alloy aged at 723 K for 2.5 h to obtain DP and annealed at 925 K for 10 min.

The first ever evidence of a DD process in a Fe-Zn system observed via transmission electron microscopy is shown in Figure 5. Like in the case of SEM, the places where previous Γ phases existed and so-called “ghost lines” reflecting the location of the receding RF are visible; however, this is not the same picture as that previously observed using light [5,9] or scanning electron microscopy [3,4]. This difference can be explained by the fact that “ghost lines” and “ghost images” are visible on the light or scanning electron microscopy images only after appropriate etching with the composition-sensitive etchant. In the case of TEM, the sample preparation does not involve etching. To reveal such peculiarities it is necessary to imagine thin foils under various inclinations in regard to the incident electron beam, which is not easy going task. On the other hand, wide tilting in different directions also eliminates the possibility that the observed “ghost image” is an artefact coming from the surface intersection of the colony of discontinuous precipitates with the top and bottom of the thin foil.

The solute concentration profile reflecting the changes of chemistry after the DD reaction is described by the following equation derived by Zieba and Pawlowski [11]:

(1) $$x\left(y\right)=A\cdot \sin\;h\;\left(zy\lambda_{\alpha }\right)+B\cdot \cos\;h\;\left(zy\lambda_{\alpha }\right)+\frac{a}{p^{2}-z^{2}}\;\cos\;h\;\left(py\lambda_{\alpha }\right)-\frac{b}{p^{2}-z^{2}}\;\sin\;h\;\left(py\lambda_{\alpha }\right)+x_{o}$$

where

$$p={\left(\frac{v_{DP}}{s\delta D_{b}}\right)}^{\frac{1}{2}}\;,\;z={\left(\frac{v_{DD}}{s\delta D_{b}}\right)}^{\frac{1}{2}},\;A=-B\cdot tan\;h\;\left(\frac{z\lambda_{\alpha }}{2}\right)$$

$$B=x^{*}-x_{o}-\frac{a}{p^{2}-z^{2}}\;,\;a=\left(x_{o}-x_{i}\right)z^{2},\;\;\;b=a\cdot tan\;h\left(\frac{p\lambda_{\alpha }}{2}\right)$$

where *x_o_* is the initial solute concentration in the alloy, *x_i_* is the solute concentration in the *α* lamella at the *α*/Γ interface, x* is the solute concentration in the newly formed solid solution at the tip of the Γ lamella, *v_DP_* and *v_DD_* are the rates of DP and DD, respectively, *s* is the segregation factor, *δ* is the grain boundary width, *D_b_* is the grain boundary diffusion coefficient, *λ_α_* is the thickness of the *α* lamella and *y* is a normalized co-ordinate measured from the edge of the Fe_3_Zn_10_ phase in the direction perpendicular to the *α* lamella. 

In order to use Equation (1), it is necessary to know parameter *p* for describing the kinetics of the *DP* reaction, which is related to Cahn′s parameter *C* and the thickness of the α lamella with the simple relationship *p* = $\sqrt{C}$/λ_α_. The required data for calculation were taken from direct measurements of Zn profiles across the *α* lamellae [7], which was performed after ageing at a temperature range from 623 K to 773 K (Table 1). With values of *p*, it was possible to calculate parameter z for describing the kinetics of the *DD* reaction for various values of x* with the following equation:

(2) $$\lambda_{\Gamma }x_{\Gamma }=\frac{2}{z}⎣\left(x^{*}-x_{o}-\frac{z^{2}\;\left(x_{o}-x_{i}\right)}{p^{2}-z^{2}}\right)\tan\;h\;\left(z\lambda_{\alpha }/2\right)+\frac{pz\;\left(x_{o}-x_{i}\right)\;\tan\;h\left(\frac{p\lambda_{\alpha }}{2}\right)}{p^{2}-z^{2}}⎦$$

where the thickness of the Zn-rich lamella, *λ*_Γ_, is calculated from the first criterion of the applicability of the Equation (2) [11]:

(3) $$\lambda_{\Gamma }=\frac{2\left(x_{o}-x_{i}\right)\;\tan\;h\left(\frac{p\lambda_{\alpha }}{2}\right)}{px_{\beta }}$$

**Figure 6:**
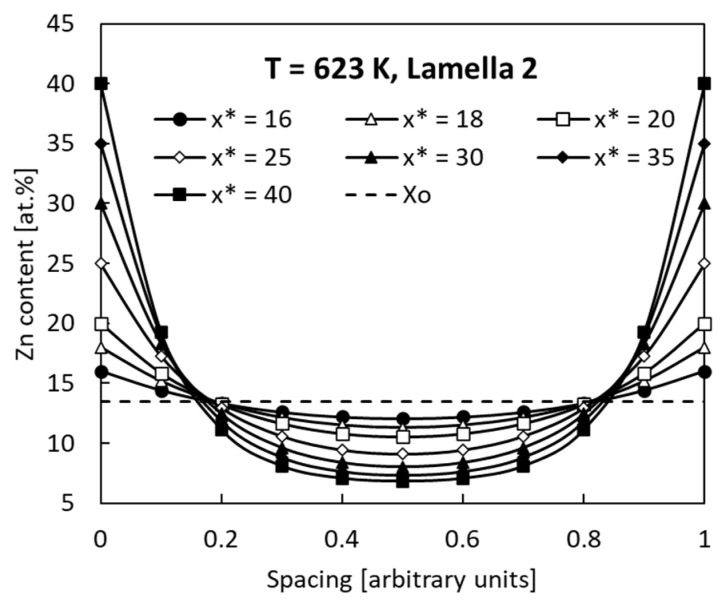
Zn concentration profiles after discontinuous dissolution for various concentrations x* (16 to 40 at. % Zn). Lamella 2 aged at 623 K (see Table 1).

**Figure 7:**
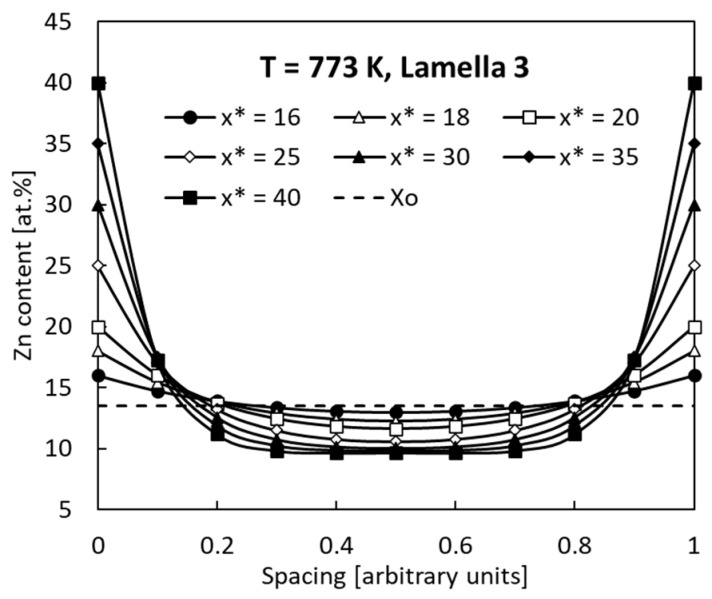
Zn concentration profiles after discontinuous dissolution for various concentrations x* (16 to 40 at. % Zn). Lamella 3 aged at 773 K (see Table 1).

Two extreme cases for the smallest and highest *p* parameters are shown in Figure 6 and Figure 7 as appropriate examples of the simulation. 

One should note that the presented curves, like all others, also satisfy the second criterion of applicability for Equation (2) as given in the following form [12]:

(4) $$x^{*}=x_{o}+\frac{a}{\left(p^{2}-z^{2}\right)}\left[1-\frac{z\;tan\;h\;\left(\frac{p\lambda_{\alpha }}{2}\right)}{p\;tan\;h\;\left(\frac{Z\lambda_{\alpha }}{2}\right)}\right]$$

All the profiles show the characteristic U shapes with a maximum at *y* = 0 and *y* = 1 which correspond to the edges of previous Γ (Fe_3_Zn_10_) lamellae. It is visible that an increase in the x* concentration results in an increase of the profile “depth” measured as the difference (x* − *x* (*y* = 0.5)) from 4.0 to 32.3 at. % Zn and from 3.0 to 30.35 at. % Zn at dissolution after ageing at 623 K and 773 K, respectively. All the profiles satisfy Equation (4), which means that the dashed horizontal line in Figure 6 and Figure 7 divide the areas above and below the *x*(*y*) curves into equal parts. As a consequence, all the x* concentrations in the range from 16 to 40 at. % Zn give correct simulation results. 

**Figure 8:**
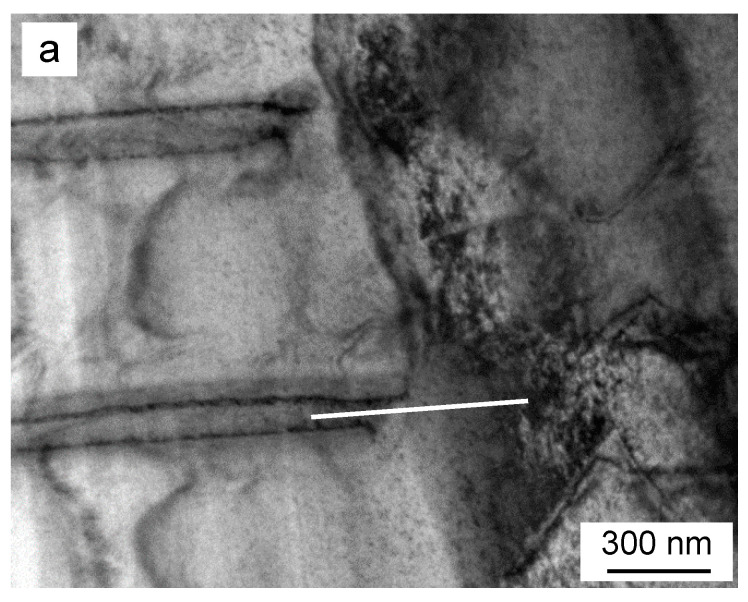

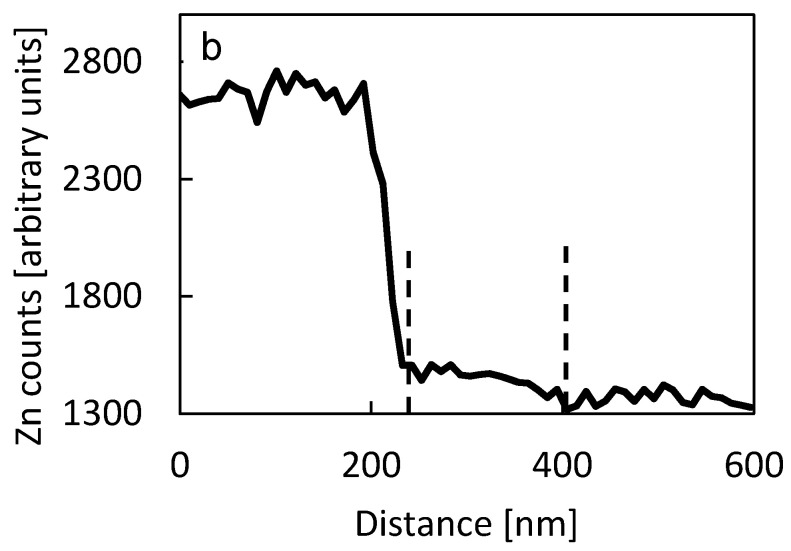
(**a**) TEM micrograph showing dissolution of the discontinuous precipitates in a Fe-13.5 at. % Zn alloy aged at 723 K for 2.5 h to obtain DP and annealed at 925 K for 10 min. (**b**) The profile of Zn taken along the solid line in Figure 8a.

The simulation procedure did not provide us with the information about time and temperature of the *DD*, which obviously influence the value of the x* concentration. Therefore, an attempt to determine the Zn content close to the tip of the Γ phase lamella being dissolved was undertaken. Figure 8a shows the receding RF of discontinuous precipitates observed using TEM. The nanochemical qualitative EDX analysis was performed along the solid line and the results obtained are presented in Figure 8b. Three areas with different Zn and Fe contents were distinguished. The highest Zn content was obviously for the Zn-rich Γ lamella. The subsequent decrease was attributed to the dissolved area close the RF where the inhomogeneity of the solid solution formed due to *DD* is the highest. The smallest Zn content is related to the area where neither *DP* nor *DD* occurred. An additional test by point-to-point quantitative EDX analysis in the area limited by the dashed lines in Figure 8b resulted in a Zn content from 30 to 15 at. % Zn depending on the distance from the RF. This clearly shows that Zn concentration just behind the tip of the receding tip of Γ lamella is time-dependent.

## 4. Conclusions

The following conclusions can be drawn from the present study:

1. The investigation of the Fe-13.5 at. % Zn alloy aged at 723 K and subsequently annealed at 925 K revealed that the dissolution process occurs in the discontinuous mode by the receding of the former reaction front of the discontinuous precipitation towards the position of the original grain boundary.
2. The *α* solid solution resulting from the discontinuous dissolution process is especially inhomogeneous. This is manifested by the “ghost images” of the places where the (Fe_3_Zn_10_) phase lamellae previously existed and “ghost lines” of the successive positions of the receding reaction front, both clearly confirmed by light and scanning electron microscopy observations and, for the first time, using transmission electron microscopy.
3. The calculations found here allow the prediction of the Zn content, where the x* left at the positions of the dissolved Γ lamellae has the largest influence on the shape of post-dissolution Zn profiles.
4. The changes of x* are time-dependent, as the largest value was found to be just behind the receding tip of the Γ lamella and decreased towards the original position of the reaction front before the dissolution process started.

## Figures and Tables

**Table 1:** Data for the calculation of Zn profiles after discontinuous dissolution (DD).

T [K]	Lamella Analysed	*x_i_* [at. % Zn]	*y* (*x* = 0.2) [at. % Zn]	*λ_α_* [nm]	C	*p* [1/m^2^]	*λ*_Γ_ [nm]
623	1	1.47	4.68	210	4.88	1.05 × 10^5^	27
	2	1.53	4.66	192	4.74	1.27 × 10^5^	24.7
	3	1.55	4.85	247	5.12	9.16 × 10^6^	31.1
	4	1.61	4.98	265	5.33	8.71 × 10^6^	32.9
	5	1.68	5.18	330	5.69	7.23 × 10^6^	40
673	1	2.37	5.33	301	4.85	7.32 × 10^6^	35.8
	2	2.54	5.56	327	5.11	1.56 × 10^5^	37.8
	3	2.21	5.14	298	4.69	7.27 × 10^6^	36.3
	4	2.17	5.53	351	5.72	7.45 × 10^6^	40.7
	5	2.27	5.43	342	5.28	6.72 × 10^6^	40.2
723	1	4.11	6.12	389	4.87	5.67 × 10^6^	39
	2	3.68	6.35	430	5.01	5.21 × 10^6^	44.8
	3	3.55	6.34	458	5.24	5.0 × 10^6^	47.8
	4	3.87	6.76	511	5.82	4.72 × 10^6^	50.1
	5	3.44	6.33	487	5.43	4.78 × 10^6^	50.9
773	1	5.76	8.33	780	6.83	3.35 × 10^6^	58.7
	2	6.03	8.39	726	6.31	4.46 × 10^6^	54
	3	5.88	8.43	797	6.92	3.3 × 10^6^	58.8
	4	5.92	8.09	589	5.42	3.95 × 10^6^	46.4
	5	5.7	7.97	601	5.56	3.92 × 10^6^	48.4

## Data Availability

Data is contained within the article.

## References

[1] Speich G.R. (1968). Cellular precipitation in Fe-Zn alloys. Trans. AIME.

[2] Predel B., Frebel M. (1972). Zur Kinetik der feinlamellaren diskontinuierlichen Ausscheidung in -Mischkristallen des Systems Eisen-Zink. Arch. Eisenhüttenwes.

[3] Chuang T.H., Gust R.A.F.W., Predel B. (1989). Drei diskontinuierliche Festkörperreaktionen in einer -Fe-13.5 at. % Zn-Legierung. Z. Met..

[4] Gupta S.P. (2001). A comparative study of the kinetics of interface diffusion controlled transformations in Fe-Zn alloys. Can. Metall. Q..

[5] Nakkalil R., Gupta S.P. (1989). Kinetics of discontinuous dissolution in an Fe-20 wt.% Zn alloy. Z. Met..

[6] Petermann J., Hornbogen E. (1968). Drei Mechanismem der Ausscheidung in Blei-Natrium-Mischkristallen. Z. Met..

[7] Zięba P., Chronowski M., Morgiel J. (2020). Micro-analytical studies of discontinuous precipitation in Fe-13.5 at. % Zn alloy. Arch. Civ. Mech. Eng..

[8] Han K., Ohnuma I., Okuda K., Kainuma R. (2018). Experimental determination of phase diagram in the Zn-Fe binary system. J. Alloys Compd..

[9] Sulonen M.S. (1960). Discontinuous mode of dissolution of a phase precipitate into Cu-Cd solid solutions. Acta Met..

[10] Chronowski M., Zięba P. (2020). On the -go and -stop motion of the discontinuous precipitation front. Arch. Civ. Mech. Eng..

[11] Zięba P., Pawłowski A. (1968). Analysis of cellular dissolution model. Scr. Met..

[12] Zięba P., Gust W. (1998). Solute Concentration Profiles for Discontinuous Dissolution in Al-22 at. % Zn Alloy. Interface Sci..

